# Screening and Testing for Homologous Recombination Repair Deficiency (HRD) in Breast Cancer: an Overview of the Current Global Landscape

**DOI:** 10.1007/s11912-024-01560-3

**Published:** 2024-06-01

**Authors:** Gordon R. Daly, Sindhuja Naidoo, Mohammad Alabdulrahman, Jason McGrath, Gavin P. Dowling, Maen M. AlRawashdeh, Arnold D. K. Hill, Damir Varešlija, Leonie Young

**Affiliations:** 1 The Department of Surgery, RCSI University of Medicine and Health Sciences, Dublin, Ireland; 2 The Department of Surgery, Beaumont Hospital, Dublin, Ireland; 3 School of Pharmacy and Biomolecular Sciences, RCSI University of Medicine and Health Sciences, Dublin, Ireland; 4 Beaumont RCSI Cancer Centre, Beaumont Hospital, Dublin, Ireland

**Keywords:** Homologous Recombination Repair Deficiency, PARP inhibitors, Breast cancer, Global, Health equality

## Abstract

**Purpose of Review:**

Homologous recombination repair deficiency (HRD) increases breast cancer susceptibility and influences both prophylactic and active management of breast cancer. This review evaluates HRD testing and the therapeutic implications of HRD in a global context.

**Recent Findings:**

Ongoing research efforts have highlighted the importance of HRD beyond BRCA1/2 as a potential therapeutic target in breast cancer. However, despite the improved affordability of next-generation sequencing (NGS) and the discovery of PARP inhibitors, economic and geographical barriers in access to HRD testing and breast cancer screening do not allow all patients to benefit from the personalized treatment approach they provide.

**Summary:**

Advancements in HRD testing modalities and targeted therapeutics enable tailored breast cancer management. However, inequalities in access to testing and optimized treatments are contributing to widening health disparities globally.

## Introduction

Homologous recombination repair deficiency (HRD) significantly increases breast cancer predisposition and tumor aggressiveness. However, through ongoing, extensive research into its mechanism, it has an evolving role as a provider of therapeutic targets. HRD represents a pivotal functional defect within the complex landscape of effective DNA repair, specifically in homologous recombination repair (HRR) [1]. HRD describes a cellular condition characterized by the inability to effectively repair double-stranded DNA breaks via the HRR pathway [2]. This deficiency arises from various genetic alterations, including germline or somatic mutations in key DNA genes such as BRCA1, BRCA2, and other integral genes in the HRR pathway [1, 3]. The repercussions of these mutations significantly contribute to increased genomic instability, with particularly profound implications in breast cancer [3].

**Fig. 1:**
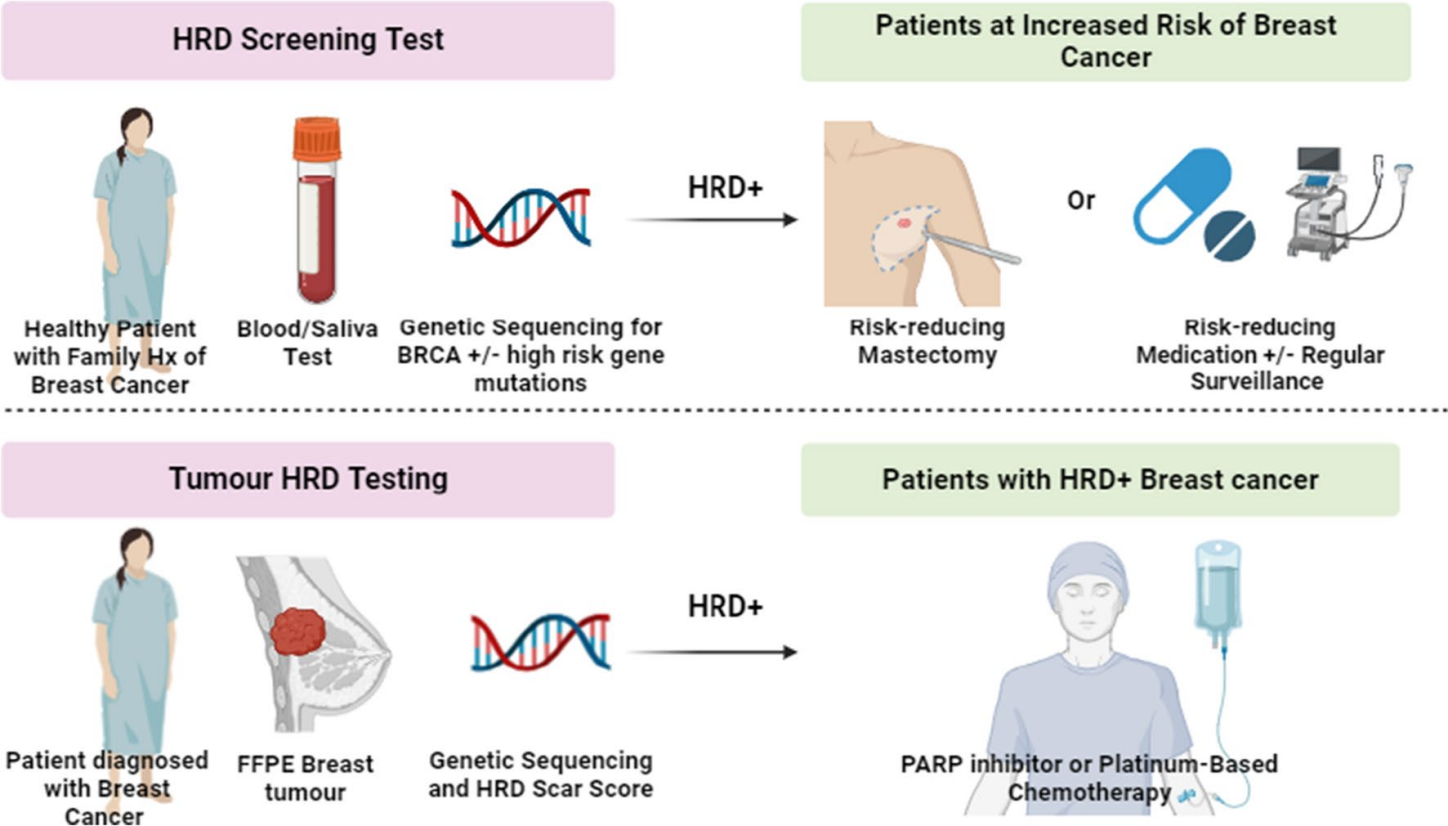
Summary of HRD screening and testing. Healthy patients with a family risk of hereditary breast cancer may undergo genetic testing for HRD. If HRD is detected, management options include risk-reducing mastectomy (RRM), risk-reducing medications or regular monitoring. RRMs include selective estrogen receptor modulators (SERMs) and aromatase inhibitors (AIs). Magnetic resonance imaging (MRI) is advised one year after commencing a RRM to establish a baseline, with no additional imaging recommended in the absence of clinical findings. Patients unwilling to take RRMs should be monitored with yearly MRIs/ Ultrasound (US) and 6-monthly/yearly physical examinations. Patients diagnosed with an HRD + breast cancer may benefit from PARP inhibitor treatment or a platinum-based chemotherapy regimen. Created from https://www.biorender.com/

HRD positivity influences both prophylactic measures when detected in healthy individuals and treatment options for patients diagnosed with HRD-positive cancer. Summary in Fig. 1. Although HRD status is clinically significant in various cancers, its notably higher prevalence in breast cancer underscores its importance as a key factor in the care of both primary and metastatic disease [1].

Beyond BRCA1/2, a diverse array of genetic mutations can also cause HRD, providing multiple targets for tailored management. For instance, tmutation of the RAD51c gene contributes to a 20% lifetime risk of breast cancer. Moreover, the PALB2 gene is associated with a higher risk, ranging from 40–60% [4]. These intricate genetic variations highlight the complexity of HRD status in breast cancer and the importance of comprehensive testing for HRD beyond just BRCA1 and BRCA2.

However, discrepancies in the cost and availability of testing across countries, along with ethical dilemmas surrounding HRD testing and its significant psychological impact on individuals, present both great opportunities and difficult challenges for patients and clinicians. This literature review outlines the various modalities, guidelines and treatment implications of screening and testing for HRD in a global context and discusses the nuanced ethical, social, and economic implications that vary between international jurisdictions.

## Methods of HRD Testing

Effective HRD detection has a pivotal role in both accurately selecting breast cancer patients to avail of targeted therapies and in identifying the patients with an inherited risk of breast cancer who will benefit from prophylactic measures. Methods of HRD testing have undergone significant technological advancements, playing a crucial role in research on HRD-positive breast cancer and the development of targeted HRD-based therapeutic treatments. Nonetheless, measuring HRD involves several diagnostic challenges. One primary issue is that the definition of HRD varies across different testing methods and commercial entities. Additionally, testing methods often rely on expensive next-generation sequencing (NGS) or labor-intensive functional assays. Despite these challenges, the increased affordability of NGS and advancements functional assays have made testing more feasible in recent years.

Recent research has introduced several effective methods for HRD and BRCA screening, particularly in patients with a family history of breast cancer. A significant breakthrough is the development of the Bayesian probabilistic model, BRCAPRO, noted for its accuracy in identifying individuals for BRCA1 and BRCA2 mutation testing. This model outperforms traditional clinical scoring methods by incorporating breast cancer pathology data, such as tumor grade and hormone receptor status, thereby enhancing its precision [5]. Furthermore, morpho-clinical parameters, notably estrogen receptor (ER) negativity and poor tumor differentiation, have shown effectiveness in detecting BRCA1 mutations. This approach is particularly valuable in instances where there is a weak or absent family history of these cancers [6].

Traditional screening methods have primarily relied on Sanger sequencing of a multigene panel or a Multiplex Ligation-dependent Probe Amplification (MLPA) assay for detecting copy number variations in blood or saliva samples. While these techniques are accurate, they are less cost-effective compared to NGS [7, 8]. High-resolution melting (HRM) analysis has also gained prominence as a promising rapid screening technique for identifying BRCA1/2 mutations. This method is adept at distinguishing genetic variants in PCR products [9]. Furthermore, population-based studies indicate that a significant portion of breast cancer cases, irrespective of subtype, may exhibit defects in the HRR pathway. Consequently, there is a recommendation for population-based genetic testing to achieve a broader identification of individuals at risk [10].

Several commercially available sequencing tests for HRD in diagnosed breast cancer, such as FoundationOne CDx and MyChoice, offer insights into the genetic underpinning of the disease [11]. Typically, these tests involve whole genome sequencing (WGS) or whole exome sequencing (WES) performed on formalin-fixed paraffin-embedded (FFPE) tumor samples. These tests are utilized to identify mutations in BRCA1/2 or other HRD-related genes. Additionally, genomic data is utilized to calculate a ‘genomic scar score’ which is derived from parameters of DNA damage, including loss of heterozygosity (LOH), telomeric allelic imbalance (TAI), and large-scale state transitions (LST). These measures serve as indicators of genomic instability [1].

An alternative approach is the RAD51 assay, which estimates the amount of nuclear RAD51, a gene involved in HRR and crucial for template strand invasion [11]. This assay represents an effort to develop direct functional assays. However, it is one of many functional assays that face limitations rendering them less practical in a clinical setting [11]. Despite demonstrating 90% sensitivity in BRCA-deficient tumors, when refined using formalin-fixed paraffin-embedded FFPE samples, the higher costs and longer lab turnaround times associated with clinical assays undermine their effectiveness compared to WGS or WES [11]. Emerging assays, including SOPHiA Genetics DDM HRD and the AMOY HRD Focus Panel, are under development [11]. Additionally, advancements in machine learning (ML) technologies hold substantial promise for the future of HRD detection in breast cancer tumors, particularly in the analysis of histopathological specimens and NGS [12, 13].

The field of HRD testing methods is rapidly evolving, with developments focusing on becoming more cost-effective and precise. However, there is a critical need for cohesive international efforts to ensure these technologies continue progress and to become accessible to all patients, regardless of geographical location. This global approach is essential for leveraging the full potential of these technologies, ensuring equitable access to cutting-edge diagnostic tools, and ultimately improving outcomes for patients with breast cancer worldwide.

### Treatment Implications

The identification of a germline mutation in an HRD gene through screening of patients at high-risk of hereditary breast cancer plays a crucial role in determining optimal management strategies for breast cancer prevention. This includes a range of risk-reducing surgical approaches,pharmacological interventions and increased patient monitoring.

In terms of prophylactic measures, guidelines for risk-reducing mastectomy (RRM) from the European Society of Medical Oncology (ESMO), the National Institute for Health and Care Excellence (NICE), and the National Comprehensive Cancer Network (NCCN) provide crucial frameworks for individuals at increased risk of hereditary breast cancer [4, 14, 15].

ESMO particularly advocates for bilateral risk-reducing mastectomy (BRRM) as a highly effective treatment, extending its recommendations to carriers of various high-risk genes [4]. NICE places a strong emphasis on informed decision-making, facilitated by genetic counselling, and considers individual factors such as age and family history [14]. It underlines the importance of verifying family history and, if necessary, seeks consensus from a multidisciplinary team. The NCCN aligns with both ESMO and NICE in endorsing RRM as an effective risk-reduction strategy, strongly emphasizing the importance of health maintenance post-RRM [4]. Risk-reducing medications (RRMeds), such as selective estrogen receptor modulators (SERMs) and aromatase inhibitors (AIs), are an alternative for women who postpone or decline RRMs [4]. A network meta-analysis by Mocellin et al. found, that in women with above average risk, both AIs and SERMs reduced breast cancer incidence [16]. They concluded SERMs were less effective than AIs and are associated with a risk of endometrial cancer and venous thromboembolic disease [16]. However, even in patients with diagnosed breast cancer, adherence to endocrine therapy presents a major challenge [17]. The cost, side effects and the long-term nature of endocrine therapy are among the causes of poor adherence [18]. Strategies for promoting adherence include lowering medication cost and psychosocial and reminder counselling.

It is important to note both RRM and conservative management require adequate follow-up. While yearly MRIs/ Ultrasound and 6-monthly/yearly physical examinations (depending on mutation type) are advised for conservative management, an MRI one year post RRM to establish a baseline may be sufficient in the absence of further clinical findings [4].

In a study which included 2,677 mutation carriers, Metcalfe et al. examined the international variation in subsequent management of BRCA1/2 mutation carriers [19]. 41 centers across nine countries, including Austria, Canada, France, Israel, Italy, Norway, Holland, Poland, and the US were included [19]. Individuals who received genetic test results indicating a BRCA1/2 mutation were subsequently questioned about the preventive practices they adopted following receipt of their results [19]. This study highlights that management post genetic testing, in reality, is dictated by a combination of guidelines, patient preferences and the availability of resources.

Therapeutically targeting HRD in breast cancer leverages genetic abnormalities within cancer cells to induce cell death. Poly (ADP-ribose) polymerase inhibitors (PARPis) have become a crucial treatment for breast cancer with BRCA mutations. The primary action of PARPis is their effectiveness against tumors exhibiting HRD, utilizing a process called synthetic lethality [20]. Synthetic lethality arises when the loss of a single gene is tolerable for cell survival, but the concurrent disruption of two genes leads to cell death [20]. PARPis inhibit PARP by competing with NAD + at the catalytic domain (CAT) of PARP, thus impeding PARP's catalytic activity and the formation of PAR polymers [21]. These actions compromise the cellular capacity to repair DNA single-strand breaks (SSBs). Furthermore, PARP inhibition can transform unrepaired SSBs into double-strand breaks (DSBs) due to replication fork collapse, a process known as the PARP trapping mechanism [21]. An additional proposed mechanism involves the trapping of PARP1 on DNA, causing substantial damage that cells with HRD are unable to repair [22].

Clinical trials like EMBRACA, OlympiA, and OlympiAD have demonstrated the efficacy of PARPis, leading to the FDA approval of talazoparib and olaparib in the treatment of breast cancer [23–25]. Summary in Table 1. Both olaparib and talazoparib are licensed for patients with BRCA-mutated or suspected germline deletions, HER2-negative locally advanced or metastatic breast cancer) [22]. By 2022, olaparib received FDA approval as an adjuvant treatment for germline BRCA-mutated or suspected deletions, HER2-negative, or high-risk early-stage breast cancer in patients who had undergone neoadjuvant or adjuvant chemotherapy [26]. In ovarian cancer, olaparib's approval was further expanded to include patients with non-BRCA HRD, as defined by the MyChoice CDx genomic instability score. This underscores the importance of identifying all HRD types in breast cancer and suggests the potential to broaden the cohort of patients receiving PARPi treatment [26].

**Table 1:** Summary of the key trials of PARP inhibitors in breast cancer

Trial	EMBRACA [23]	OlmpiAD [24]	OlympiA [25]
Population	gBRCA1/2-mutated HER2-negative locally advanced/metastatic breast cancer	gBRCA1/2 mutated HER2–negative metastatic breast cancer	gBRCA1/2 mutated HER2–negative, early breast cancer
Intervention	Talazoparib	Olaparib	Olaparib
Comparison	Physician’s choice of chemotherapy*	Standard Therapy**	Placebo
Outcome	Median PFS in talazoparib group was 8.6 months vs. 5.6 months in control (HR 0.54, 95% CI 0.55–0.41, *P* < 0.001)	Median PFS in olaparib group was 7 months vs. 4 months in control (HR 0.58, 95% C.I 0.43–0.80, *P* < 0.001)	3-year invasive DFS rate in olaparib group was 85.9% vs. 77.1% control, HR 0.58, 95% CI 0.41–0.82, *P* < 0.001)
Adverse events of intervention	Nausea (48.6%), vomiting (24.8%) fatigue (50.3%), headache (32.5%), anaemia (52.8%), neutropenia (34.6%), leukopenia (17.1%)	Nausea (58%); vomiting (29.8%); fatigue 28.8%; anaemia (40%); headache (20%); neutropenia (27.3%); leukopenia (16.1%)	Nausea, fatigue, anaemia, vomiting, headache, neutropenia, leukopenia, AML

* Capecitabine, eribulin, gemcitabine, or vinorelbine in continuous 21-day cycles

**Capecitabine: 2500 mg/sqm orally daily for 14 days, repeated every 21 days; eribulin mesylate: 1.4 mg/sqm intravenously on days 1 and 8, repeated every 21 days; Vinorelbine: 30 mg/sqm intravenously on days 1 and 8, repeated every 21 days

gBRCA, germline BRCA; PFS, progression-free survival; DFS, disease-free survival; HR, 95% CI, hazard ratio, 95% confidence interval; AML, acute myeloid leukaemia.

NCT03344965 is an ongoing phase 2 study that aims to extend the use of olaparib monotherapy in metastatic breast cancer patients with germline or somatic mutations in DNA repair genes, with completion expected in December 2024 [27]. The genes under investigation include BRCA1/2, CHEK2, ATM, PALB2, RAD51, BRIP1, and NBN [27]. Other clinical trials are exploring alternative PARPis in non-BRCA HRD. The PETRA trial, a phase 1/2 study, is examining AZD5305 in several advanced cancers, including triple-negative breast cancer (TNBC), with mutations in BRCA1/2, PALB2, or RAD51C/D [28]. These mutations are amongst the most common recognized in HRD [26]. AZD5305 is noted for being a potent, highly selective PARP1 inhibitor, significantly more selective for PARP1 than PARP2, and particularly effective in HRD [26].

Additionally, the University of California, San Francisco is conducting a phase 1 clinical trial (NCT05694715) to assess the impact of the combination of niraparib and irinotecan on managing solid tumors with HRD-positivity [29]. This trial includes individuals with metastatic solid tumors and mutations in BRCA1/2, ATM, or PALB2 [29]. The combination of niraparib and irinotecan has garnered considerable interest, and the dose–response results from phase II will inform the ongoing NCI ComboMatch trial [29].

In the rapidly changing field of HRD research, technological advancements have been pivotal in both understanding HRD-positive breast cancer and developing targeted therapeutic strategies. Presently, small molecule inhibitors aimed at key proteins such as Polymerase theta (Polθ), RAD51 homolog 1 (RAD51), ubiquitin carboxyl-terminal hydrolase 1 (USP1), poly (ADP-Ribose) glycohydrolase (PARG), and Werner syndrome helicase (WRN) are under clinical investigation [30]. Notably, synergistic effects have been observed in BRCA-deficient models with combinations of PARPis and Polθ inhibitors, indicating potential new therapeutic pathways [30]. The commencement of the first in-human study (NCT05787587) of IDE161 (a PARG inhibitor) as monotherapy in solid HRD breast and ovarian tumors underscores the ongoing commitment to advancing targeted HRD-based treatments [30].

The advantage of HRD screening lies in its ability to identify high-risk patients who may benefit from an RRM and/or regular breast screenings. When applied to confirmed cases of breast cancer, HRD testing facilitates the identification of specific targets for personalized management. This evolving understanding of HRD leads to more individualized and effective patient treatments. However, the accessibility to such testing and the availability of these often expensive treatments are not universal, contributing to the increasing healthcare disparity between developed and developing countries, as well as among different socioeconomic groups.

### Cost Effectiveness of HRD Screening

The cost-effectiveness of HRD screening and its implications for prophylactic management involve a significant financial consideration. The feasibility of HRD testing and the affordability of subsequent treatments exhibit substantial variation across the globe, compounded by differences in healthcare systems, insurance approval processes, and pricing structures.

A study from Switzerland highlighted the potential cost-effectiveness of more invasive prophylactic strategies, such as prophylactic bilateral mastectomy, for BRCA1/2 mutation carriers, suggesting that such measures could be economically viable in the long term [31]. However, access disparities remain a challenge, with some individuals struggling to obtain necessary healthcare due to insurance constraints or personal coverage limitations [31]. In the United States, financial hurdles, including lack of insurance, insufficient coverage, or direct costs of testing, contribute to the underutilization of BRCA screening, particularly affecting vulnerable populations [32, 33]. This issue is exacerbated in developing countries, where the costs of genetic testing and associated travel expenses pose significant barriers [33].

Xi et al. conducted a scoping review focused on the economic evaluations of predictive genetic testing, including studies on BRCA1/BRCA2 testing for hereditary breast and ovarian cancer (HBOC) [34]. This review identified three primary cost components: the cost of testing, prevention, the disease management. The majority of the reviewed studies concluded that genetic testing is cost-effective compared to no testing, with some suggesting that multigene testing could be economically viable under certain conditions. However, there was variability in the cost-effectiveness of different gene panels and the overall savings associated with population-based testing [34].

Koldehoff et al. performed a systematic review analyzing the cost-effectiveness of targeted genetic screening for breast cancer [35]. Their findings indicated variable cost-utility ratios from a payer’s perspective, influenced by factors such as discount rates, the choice of prophylactic surgery, and mutation penetrance. Probabilistic analyses showed a high likelihood of cost-effectiveness for BRCA testing in several studies, though results varied for multigene tests, highlighting the economic complexity of genetic testing for breast cancer susceptibility [35].

These studies underscore the economic benefits of genetic testing for breast cancer and susceptibility, with variations in the optimal testing strategies influenced by numerous factors. The economic implications are complex, requiring tailored considerations to enhance the accessibility and affordability of HRD screening globally. These findings stress the importance of developing and implementing strategies that address the economic challenges associated with genetic testing, ensuring equitable access to life-saving interventions.

### Ethical and social implications of HRD screening

Screening for HRD presents significant ethical challenges, particularly in cases where a cancer syndrome or familial inheritance pattern is suspected [36]. Clinicians must understand that knowledge of an individual's mutational status can profoundly affect their family life, personal finances, and potentially, their psychosocial well-being [36]. The benefits of disclosing genetic mutations must be carefully weighed against the ethical and social implications stemming from the increased availability of HRD testing.

A study analyzing 50 subjects who underwent BRCA1/2 gene testing for suspected BRCA gene mutations found that genetic testing facilitated easier communication within families [36]. Patients who had undergone testing were more inclined to discuss the possibility of genetic testing with their family, in contrast to 50% of the non-genetic testing group who deemed it inappropriate to discuss such matters with relatives [36]. This study highlighted the role of good communication in enhancing family cohesion and influencing the decision to undergo genetic testing for BRCA mutations [36]. Nevertheless, it is essential to consider the impact of cultural norms and the openness of discussions within families.

Conversely, the presence of a stable partner was observed to discourage patients from undergoing genetic testing to determine their mutational status [36]. This reluctance aligns with findings from studies indicating the significant distress associated with BRCA testing, leading many women to prefer not disclosing decisions about testing and oncogene counselling [36, 37]. The concept of 'geneticization' raises deep questions about responsibility, education, and the societal impacts of HRD testing. Mediation analyses on a sample of 178 women undergoing their first genetic counselling session for breast/ovarian cancer showed that cancer-related worry and risk perception based on genetic counselling were associated with increased levels of depression and anxiety [38]. Further analysis revealed that cancer-related worry, but not risk perception, was heightened among those with a prior cancer diagnosis [38]. Additionally, the number of family members affected by cancer was linked to increased cancer-related worry and risk perception [38]. Understanding patients' genetic health literacy, risk perception, and beliefs about disease and prevention is therefore paramount [38].

Disclosing information about cancer susceptibility carries significant implications for patients and their families [39]. A study found that women with higher depression scores reported elevated risk estimates of developing breast cancer [39]. As depression levels increased, so did the intensity and frequency of cancer-related worry and risk perception [39]. While genetic testing aims to enable preventative measures, the psychological impact of such tests and their ability to affect various aspects of a patient's life must be considered [39].

The ESMO recommendations highlight the importance of post-test genetic counselling, personalized risk management, and specialized high-risk clinics, emphasizing the need for awareness and accessibility of testing for at-risk relatives [4]. The psychological impact of genetic counselling and testing post-cancer diagnosis varies, with some reporting distress while others find it acceptable, highlighting its role in informed treatment decisions. Larger, prospective studies are encouraged to provide conclusive evidence [40–42]. However, the right to undergo genetic testing raises discussions about access, cost, and familial rights [39]. The 'right not to know' is contested between individual autonomy and societal obligations, with concerns about genetic discrimination posing significant challenges, especially in relation to health insurance [43].

Research by Tercyak et al. on quality of life after contralateral prophylactic mastectomy indicates that, in the first year post-surgery, patients' quality of life and distress levels do not significantly differ from those opting for alternative treatments [44]. A 10-year follow-up study by Frost et al. suggests that despite negative impacts on body image and relationships, a significant majority express satisfaction with their decision [45].

Navigating the multifaceted ethical landscape of HRD screening requires balancing individual liberties, societal responsibilities, and the evolving medical knowledge landscape. Adequate patient counselling on the benefits and pitfalls of HRD testing at an individual level is crucial. This approach empowers patients to be involved in their care and make decisions that are right for them.

### Global implications of HRD screening and testing

#### Europe

The global landscape of HRD screening and testing is marked by diverse approaches and guidelines, with Europe playing a significant role in shaping these practices. ESMO has set forth comprehensive recommendations for the management of hereditary breast cancer, highlighting the critical role of genetic testing. ESMO's guidelines advocate for the use of multigene panels in individuals with a significant family history of cancer, acknowledging that reliance solely on family history for testing may overlook nearly half of the carriers of HBOC syndrome genes [4].

Further, consensus statements from Europe and the UK have provided additional guidance on the genes that should be included in screening/testing panels [46, 47]. The European consensus statement advises that HRD testing should extend beyond BRCA1/2 genes when resources permit. It suggests that the inclusion of additional genes should be tailored based on the patient's history, underlining the necessity of considering the psychological impacts of genetic testing [46]. This approach acknowledges the complex scenario faced by patients carrying mutations in genes of low or intermediate penetrance, who may have unrealistic expectations regarding treatment options, given that their mutations might not have clear clinical actionability [46]. These findings and recommendations are detailed in Table 2.

**Table 2:** Summary of the European expert consensus statement regarding genetic counselling, and testing of susceptibility genes for therapeutic decision-making in breast cancer [46]

1) High throughput sequencing technologies (i.e. NGS) are a sufficient testing method
2) Patients with familial history of breast cancer should not be restricted to only BRCA1/2 predictive
3) BRCA1/2, TP53 and PALB2 should all be included on a basic gene panel
4) Different populations may require different gene sets: PTEN, STK-11, ATM, CHEK2, CDH-1, MLH1, MSH2, MSH6, BRIP1, and RAD51D/C
5) 69% recommended that testing should be offered to all patients with breast cancer, and not restricted to advanced disease

In the United Kingdom, expert consensus has further refined recommendations for the clinical management of mutations identified through HRD screening, particularly those outside of the BRCA1/2 genes, in patients with a family history of breast cancer [47]. This consensus stresses the importance of including genes such as RAD51c, RAD51d, and PALB2 in the HRD screening process for these patients [47]. These recommendations reflect a tailored approach to genetic testing, aiming to enhance the detection and management of hereditary breast cancer risk while also addressing the psychological and clinical implications for the patients. The key points of this consensus are summarized in Table 3.

**Table 3:** Summary of the United Kingdom expert consensus for clinical management of non-BRCA1/2 HRD breast cancer gene mutations [47]

Recommendations for RAD51c/D carriers-
1) Include RAD51C and RAD51D on predisposition gene panel
2) RAD51C and RAD51D carriers should undergo surveillance based on an individualised risk assessment
- Carriers aged 40–49 with lifetime risk of 17–30% should be offered annual mammograms (moderate risk) followed by NHS Breast Cancer Screening Program (NHSBSP)
- Carriers aged 40–59 with a lifetime risk of > 30% but < 40% (high-risk) should be offered annual mammograms followed by the NHSBSP
- Carriers with a lifetime risk of > 40% ((very high-risk (VHR)) should be referred to VHR Breast Screening programme
3) RAD51C and RAD51D carriers with a lifetime BC risk of ≥ 30%
- Risk-reducing mastectomy (RRM) should be discussed with an individualized risk assessment
- offer alongside counselling and shared decision making
Recommendations for PALB2 carriers-
1) Breast cancer predisposition gene panel should include PALB2
2) Individualized risk assessment should determine breast surveillance for PALB2 carriers
- Carriers should be referred to NHSBSP VHR screening programme (age 25–30 years) based on risk
- RRM can be discussed with PALB2 carriers with a lifetime breast cancer risk ≥ 30%, in conjunction with an individualised risk assessment, appropriate counselling and shared decision making
Recommendations for BRIP1 carriers-
1) Breast cancer gene predisposition panel should not include BRIP1
2) BRIP1 carriers should undergo surveillance based on family history, not BRIP1 carrier status

These developments in Europe and the UK underscore a proactive and nuanced approach to HRD screening and testing. By advocating for the use of comprehensive gene panels and considering the individual patient's history and psychological well-being, these guidelines aim to improve the detection and management of hereditary cancer risk, offering a model for other regions grappling with similar challenges.

#### United States

In the United States, the landscape of HRD testing is shaped by guidelines from two expert panels: the National Comprehensive Cancer Network (NCCN) and the American Society of Breast Surgeons (ASBrS) [15, 48]. These guidelines, summarized in Tables 4 and 5, outline an approach to testing but differ in their recommendations regarding which genes to include and the criteria for patient eligibility.

**Table 4:** Summary of NCCN *Genetic and Familial High-Risk Assessment: Breast, Ovarian, and Pancreatic version 2.2024* [15]

When is HRD testing recommended?
1) In confirmed breast cancer when-
- ≤ 50 years at diagnosis
-HER2-negative breast cancer (BC)
-TNBC
-Multiple primary BC
-Lobular BC with family history (hx) of diffuse gastric cancer
-Ashkenazi Jewish heritage
-Family History of ≥ 1 close blood relative with breast cancer ≤ 50 years
-Male BC
- ≥ 3 diagnoses of BC in relatives on same side of family, including the patient themselves
2) A family history of breast cancer when-
-Patient does not have BC but has first or second-degree relatives that meet criteria above
-Patient does not meet criteria above but have a > 5% probability of BRCA1/2 Pathogenic or likely-pathogenic (P/LP) variant based on current probability models
When should HRD testing be considered?
-Personal BC history < 60yrs (discuss management options with genetic counselling team)
-A personal history of BC at any age, with ≥ 1 close blood relative having “intermediate-risk prostate cancer with intraductal/cribriform histology”
-Patient does not meet above criteria but has a 2.5–5% probability of BRCA1/2 P/PL variant based on current probability models
What HRD genes should be include on testing panel?
-BRCA1/2, CDH1, PALB2, PTEN, STK11 and TP53 only

**Table 5:** Summary of ASBrS recommendations on genetic counselling in breast cancer [48]

“Breast surgeons, genetic counsellors, and other medical professionals knowledgeable in genetic testing can provide patient education and counselling and make recommendations to their patients regarding genetic testing and arrange testing”
- Complex patient history/test should involve referral to a certified genetic counsellor/professional
- Wide variety of panels available, with different genes on various panels
- Lack of consensus regarding which genes should be tested
- Variation in degree of consensus with regard to risk and appropriate clinical management based on certain gene mutations
“Genetic testing should be made available to all patients with a personal history of breast cancer
- Genetic testing should be offered to all patients with breast cancer (new diagnosis or family history)
- Panel should include: BRCA1/2, PALB2 and genes consistent with the patient family history
- Identification of a mutation in a patient with a new diagnosis may influence management options
- Family members will be offered testing or individualized risk-reduction options
“Patients who had genetic testing previously may benefit from updated testing”
- Patients seen by a breast surgeon with genetic testing in the past indicating no pathogenic variant should be re-evaluated
- Patients with negative BRCA1/2 germline mutations should be considered for additional testing. Particularly patients with family members without pathogenic variants
- Genetic testing prior to 2014 most likely not have PALB2 or other large genomic changes in BRAC1/2 included
“Genetic testing should be made available to patients without a history of breast cancer who meet NCCN guidelines”
- Patients with a tested affected relative is more informative than testing themselves
- Unaffected family members can consider testing with pre-test counselling to discuss “uninformative negative”
- Consideration of a multigene panel if the family history is incomplete
“Variants of uncertain significance are DNA sequences that are NOT clinically actionable”
- This result needs to be considered as inconclusive
- Patient should be managed based on their risk factors
- Result should not influence management

The NCCN guidelines are noted for their specificity, offering detailed criteria for patient and tumor characteristics that warrant testing. They recommend testing for mutations in a select group of genes, including BRCA1/2, CDH1, PALB2, PTEN, STK11, and TP53 [15]. These criteria are designed to identify patients with HER2-negative disease, multifocal disease, and those with specific familial risk factors, ensuring that testing is directed towards those most likely to benefit [15].

Conversely, the ASBrS adopts a more inclusive stance, suggesting that all patients with breast cancer or a family history of the disease should be considered for testing [48]. This approach does not specify which genes should or should not be included in the testing panel, aiming to cast a wider net to capture any relevant genetic mutations that could influence patient care [48].

The divergent recommendations of the NCCN and ASBrS reflect different priorities in managing HRD testing. The NCCN's approach prioritizes efficiency and cost-effectiveness, focusing on high-risk genes to minimize the risk of over-investigation and the psychosocial impact of unnecessary testing. In contrast, the ASBrS's broader guidelines aim to ensure no patient who could benefit from HRD testing is overlooked, potentially identifying rarer genetic mutations with clinical significance.

These varying guidelines highlight the challenges in delivering equitable, tailored care within the context of genetic testing for breast cancer. A more balanced approach might be necessary for certain patient groups, where the decision to undergo HRD testing could be influenced by factors like age, stage of disease, and co-morbidities. While relevant for all subtypes, this is a particularly important consideration for older patients with early-stage, ER + /HER2- disease, where extensive efforts have been made to de-escalate treatment [49].

As the affordability of genetic testing improves, there may be greater consensus on HRD testing practices, enhancing the ability to offer adequate testing to those in need. The challenge for healthcare systems remains to balance the provision of universal access to testing with the delivery of individualized care that optimally benefits each patient.

#### Asia

The continent of Asia, with its diverse healthcare systems, presents unique challenges and strategies in effectively managing HBOC syndrome and allowing for the adequate provision of HRD testing. This review highlights the efforts and research findings in a number of Asian countries and focuses on expert consensus statements and large-scale cohort studies in India and China [50–52].

In China, despite a lower incidence rate of breast cancer compared to many countries, studies have shown that Chinese women tend to develop the disease at a younger age and with higher mutation rates than their Western counterparts [53, 54]. Since the 1990s, the incidence of breast cancer in China has risen to more than twice the international average [55]. However, the implementation of a national breast cancer screening program has encountered significant obstacles, including poor uptake of screening services, limited accessibility for rural populations, and high costs [56]. A review by Xia et al., published in The Lancet in 2023, outlines these key challenges in achieving effective breast cancer screening across the Chinese population [56]. Many Asian countries face similar challenges in implementing programs that are accessible to their entire populations [57]. Additionally, several studies have demonstrated that Asian migrants living in England and the US are less likely to engage in screening programs than the general population [58–60]. Other plausible explanations include inadequate patient education on the benefits of screening, poor clinician-patient communication or a lack of cultural acceptance to engage in screening programs [57].

In India, a 2019 collaborative effort among experts led to the publication of consensus recommendations aimed at improving the management of HBOC syndrome, characterized by BRCA1/2 gene mutations [51]. Summary in Table 6. The recommendations were developed with the Indian population in mind, covering genetic counselling, testing methods, clinical challenges, and referrals [51]. The Delphi process, a method also employed in European and American consensus meetings, was used to reach agreement among the experts on which recommendations should be included [51]. A subsequent study in northern India highlighted significant differences in genetic mutations between the local population and those from which international guidelines have been derived [61]. This finding emphasizes the necessity for further mutation profiling in India and the development of guidelines that are specific to the Indian population. Given India's population heterogeneity, there may also be a need for region-specific guidelines to effectively address these disparities [61].

**Table 6:** Summary of consensus statements relating to breast cancer adapted from the *Indian Society of Medical and Paediatric Oncology Consensus Document on Hereditary Breast and Ovarian Cancer (HBOC)* [51]

“Who should undergo genetic testing?”
- Breast cancer diagnosis at age < 45 years
- TNBC diagnosis age < 60 years
- Male breast cancer
- Breast cancer at any age > / = 1 close relative (First/second/third degree relative on same side of family) diagnosed with breast cancer
“What genetic test should be offered?”
1) Families with known mutations should be offered single site mutation testing
2) For unknown mutations- - BRCA1/2 sequencing by NGS plus multiplex ligation probe amplification (MLPA; BRCA1/2) for large genomic rearrangements (LGRs)
- Multigene panel testing: (“a representative model panel should include BRCA1, BRCA2, p53,PTEN, CDH1, PALB2, CHEK2, ATM, RAD51C, STK11, RAD51D, BRIP1,MLH1, MSH2, MSH6, PMS2) + MLPA (BRCA1/2) for LGRs
“What risk-reduction approaches should be offered to affected individuals?”
- Contralateral prophylactic mastectomy
- Bilateral mastectomy can be offered to patients with a known germline BRCA1/2 mutation and if there is a previous history of BC
“What risk-reduction approaches should be offered to unaffected mutation carriers?” BRCA1/2 mutation:
- Lifestyle modifications
- Avoid hormone replacement therapy (HRT)
- Encourage breast feeding
- Offer BRCA carriers prophylactic bilateral mastectomy
- Offer BC surveillance to BRCA carriers who don’t have/delay surgical risk-reduction
Breast Cancer Screening:
- Breast awareness from age 18 years
- Clinical breast examination (CBE) every 6–12 months is recommended from the age of 25 years or 10 years before youngest BC diagnosis
- Annual screening MRI (days 7–15 of menstrual cycle) should be commenced from the age of 25 with the addition of annual mammography from age of 30
- Chemoprevention: use of tamoxifen may be considered, however, the level of evidence is weak. Use tamoxifen only for BRCA2 tumors or if the first cancer was ER positive
Surveillance in male previvors:
- There are no proven risk-reducing surgical options for men
- Monthly breast self-examination starting at age 35 years
- Clinical breast examination every 12 months starting at age: 35 years
“When should poly (ADP-ribose) polymerase inhibitors be used?
Talazoparib is indicated for:
- Deleterious/suspected gBRCA-mutated
- HER2-negative locally advanced or metastatic BC

Another important consideration is that the clinical impact of HRD testing is constrained by patients’ ability to access PARPis. Both licensing and reimbursement of olaparib and talazoparib is greatly varied between Asian healthcare systems. For example, in India, talazoparib is not advertised but may be imported at the expense of the patients [62]. In Korea, it is available but not reimbursed [62]. In China, Indonesia and Japan, talazoparib is not yet licensed, unlike olaparib which is currently available [62]. While olaparib is not covered by government funding in Hong Kong, a means tested ‘safety net’ fund exists for those who meet the financial threshold [63]. Another factor that adds complexity in analysing Asian healthcare standards is that breast cancer data is not available for many healthcare systems in poorer Asian countries. However, previous large cohort studies have reported worse breast cancer outcomes amongst less affluent Asian ethnicities, especially amongst minority groups, suggesting significant discrepancies in treatments provided between regions exist [64].

These observations underscore the critical need for localized approaches in managing HBOC syndrome and HRD testing. Also, improved patient education and supports to encourage engagement in the testing that is available must be emphasized. The varied genetic profiles and systemic healthcare challenges in these countries highlight the importance of developing and implementing screening and treatment guidelines that are tailored to the specific needs and contexts of their populations.

#### Africa

In Africa, a continent marked by its vast diversity, the study and understanding of breast cancer genetics, including HRD testing, remain limited. Reportedly, only nine of the 54 countries in Africa having researched the genes involved in breast cancer [65]. The access to genetic screening and HRD testing varies significantly across the continent, reflecting disparities in healthcare resources and infrastructure. Moreover, the biology of breast cancer and hereditary patterns in many African populations show notable differences from those observed globally. Despite Africa and Asia having the lowest rates of breast cancer, African and African American women tend to develop the disease almost a decade earlier than Caucasian women and are more likely to be diagnosed with aggressive TNBC [65, 66].

The urbanization of African countries and the adoption of Western lifestyles are contributing to a projected doubling of the breast cancer burden in Sub-Saharan Africa between 2012 and 2030 [66]. In South Africa, public screening for somatic BRCA1/2 mutations is available for high-risk patients, but such screening programs are absent in many other African countries with less well-funded healthcare systems [67]. Additionally, genetic screening for breast cancer in South Africa has faced criticism for being tailored to the minority white population, underscoring the need for more inclusive and representative screening programs.

Research into the genetic variance of breast cancer in African populations is scarce, with the majority of relevant data coming from studies conducted in the US on women of African heritage [68]. Most existing guidelines are based on mutational data primarily from Caucasian women, highlighting a significant gap in representation and understanding [68].

A study by Biancollela et al. characterized BRCA1/2 mutations in 51 Burkinabe women, identifying pathogenic variants significantly different from those in the gnomAD database for African women [69]. The discovery of notable differences in a relatively small sample size suggests that the current data on HRD in breast cancer poorly represents African women and fails to capture the heterogeneity of African populations.

In Kumasi, Ghana, Amankwaa-Frempong et al. conducted a study that demonstrated the feasibility of BRCA testing in Ghana by successfully extracting and genotyping DNA for three common BRCA mutations from breast cancer tumor tissue of 521 women with a family history of breast cancer [70]. Despite acknowledging significant barriers such as specimen acquisition, transport, and genotyping expenses, this study highlighted the potential for widespread BRCA testing in Africa [70]. However, access to essential systemic breast cancer treatments remains a challenge in many African countries [71, 72]. Currently, there is a paucity of data regarding the availability of PARPis in African countries, however, given their high cost and difficult access to genetic testing, it is probable that access remains poor across the continent.

The efforts in countries like Ghana and Burkina Faso illustrate the potential for comprehensive genetic profiling and the importance of international collaboration to make technology for such testing more affordable. Ensuring broader access to personalized care, akin to that available in developed countries, could significantly impact the management and treatment of breast cancer in African women. An emphasis must be placed on improving the affordability of essential treatments to make the results of HRD testing clinically actionable.

## Conclusion

The role of detecting homologous recombination repair deficiency (HRD) is increasingly pivotal in the management of breast cancer and in informing prophylactic treatment options for high-risk patients identified through screening. Although there is consensus on the importance of extending genetic testing beyond the BRCA1/2 genes, the decision on which additional genes to include and which patients should be screened remains a matter of debate. As the approach to breast cancer care shifts towards more personalized treatments, the disparity in global access to advanced diagnostic tools and optimal treatments becomes more pronounced. Despite the advancements in making genetic testing and targeted therapies more affordable, there remains a critical need to enhance efforts toward ensuring equitable access to the benefits of HRD screening and testing. Bridging this gap is essential not only for improving patient outcomes but also for moving towards a more inclusive and fair approach to cancer care worldwide.
